# Blood count changes in malaria patients according to blood groups (ABO/Rh) and sickle cell trait

**DOI:** 10.1186/s12936-024-04886-2

**Published:** 2024-04-29

**Authors:** Euclides N. M. Sacomboio, Santo D. Zua, Adelino T. Tchivango, António D. Pululu, Adilson C. D. Caumba, Adelina B. M. Paciência, Danilson V. Sati, Sabina G. Agostinho, Yolanda S. Agostinho, Fernando G. Mazanga, Neusa B. Ntambo, Cruz S. Sebastião, Joana P. Paixão, Joana Morais

**Affiliations:** 1 Instituto de Ciências de Saúde da Universidade Agostinho Neto (ICISA/UAN), Luanda, Angola; 2 Instituto Superior de Ciências de Saúde/Universidade Católica de Angola (ISCS/UCAN), Luanda, Angola; 3 Centro de Formação em Saúde (CFS) da Clinica Multiperfil, Luanda, Angola; 4 Instituto Politécnico de Malanje da Universidade Rainha Njinga A Mbande (IPM/URNM), Malanje, Angola; 5 Instituto Nacional de Investigação em Saúde (INIS), Luanda, Angola; 6 Centro de Investigação em Saúde de Angola (CISA), Caxito, Angola; 7 Faculdade de Medicina, Universidade Agostinho Neto, Luanda, Angola

**Keywords:** Blood count, Blood group, Sickle cell trait, Malaria

## Abstract

**Introduction:**

Introduction: Malaria continues to be the leading cause of hospitalization and death in Angola, a country in sub- Saharan Africa. In 2023, in the first quarter, 2,744,682 cases were registered, and of these 2,673 patients died due to malaria disease. Previous studies have shown that the ABO blood group can affect the progression of malaria to severe conditions after *P. falciparum* infection, while the sickle cell gene offers relative protection.

**Objective:**

We investigated changes in the blood count according to blood groups (ABO/Rh) and sickle cell trait in patients with malaria in Luanda, capital of Angola.

**Methodology:**

This was a longitudinal, prospective and observational study with 198 patients hospitalized for malaria.

**Results:**

Of the 198 patients studied, 13(6.6%) were ABRh(+), 4(2.0%) were ARh(-), 49(24.7%) were ARh(+), 42(21, 2%) were BRh (+), 5(2.5%) were ORh(-) and 85(42.9%) were ORh(+). For sickle cell trait, 145(73.2%) were AA, 37(18.7%) were AS and 16(8.1%) were SS. No statistical relationship was observed between age group, sex, parasitemia, clinical picture, hematocrit, MCV, HCM, MCHC, leukocytes, NEUT, LINF and PTL values with blood groups (p<0.05), but there was a relationship between values of hemoglobin and ABO/Rh blood groups (p>0.05). There was no relationship between age, parasitemia, clinical condition, MCV, HCM and MCHC values, leukocytes, NEUT and LINF with sickle cell trait (p<0.05), but there was a relationship between sex, hemoglobin and PTL and sickle cell values. sickle cell trait (p>0.05).

**Conclusion:**

It is imperative to differentiate patients with malaria based on blood groups and sickle cell trait, taking into account mainly the blood count parameters that demonstrate that there are patients who, depending on blood group or sickle cell trait, may react weakly to malaria infection regardless of the degree of parasitemia and medical prognosis.

## Background

Malaria remains the leading cause of hospitalization and death in Angola. In 2022, around 9,211,346 cases of the disease were reported and 12,485 patients died from the disease, which is an increase of 41,886 cases compared to the year 2011 when there were fewer records of cases. It is suspected that because of attention to COVID-19, many cases ended up being underreported. In the first quarter of 2023, 2,744,682 cases were registered and of these 2673 patients died due to the disease [1, 2].

Several genetic variants or polymorphisms of red blood cells have been identified as cofactors that can make humans relatively more susceptible or resistant to *Plasmodium falciparum* and affect clinical outcomes. Many studies have shown that the interaction between the ABO blood group and the infection by *P. falciparum* can increase or decrease the severity of the disease, where it seems that individuals from blood groups A, B, and AB are more susceptible to the severity of malaria compared to those from blood group O [3–5].

Studies published so far demonstrate that the ABO blood group can affect the progression of malaria to serious situations after infection with *P. falciparum*, since some groups, such as the ABO blood group, delay the clearance of parasitized red blood cells (pRBCs) promoting the formation of rosettes and cytoadhesion, while other groups such as blood group O increase the clearance of pGVs red blood cells by reducing rosette formation and cytoadhesion [6–8].

Sickle cell anaemia is a serious public health problem, mainly present in tropical countries, especially in sub-Saharan Africa, and the World Health Organization (WHO) estimates that 300,000 children are born with sickle cell anaemia each year, 75% of which are in sub-Saharan Africa [9].

Other studies proved that the (SS) gene does not protect against infection by the malaria parasite, but prevents the establishment of the disease after infection, which demonstrates that the sickle cell gene offers relative protection against malaria, it can be expected that the protection is at least as effective in the homozygous state (SS) [10], however, clinical experience has shown that it is more dangerous, as malaria not only worsens pre-existing anaemia in SS patients to the point of becoming life-threatening but also abnormal splenic function in patients with sickle cell anaemia which makes it difficult to eliminate parasitized red blood cells and for this reason in African countries, malaria contributes substantially to the early mortality of patients with sickle cell anaemia [10, 11].

*Plasmodium falciparum* infection is usually fatal in individuals with sickle cell anaemia (HbSS), as protection against infection appears to operate in a dose-dependent manner with HbS, therefore, individuals with HbSS have an even lower risk of infection than those with HbAS [11]. Although sickle cell disease (SCD) is primarily a disease of red blood cells, both leukocytes and thrombocytes are equally affected, as in malarial infection, and are known to cause sickle cell crises through vaso-occlusion [12].

Studies developed by our research team in Angola have shown that non-O blood groups appear to be important biological factors for SARS-CoV-2 infection and the risk of developing cardiovascular disease after or during exposure to SARS-CoV-2 [13]. HIV infection seems to be common in ORh + individuals, where alterations in the blood count occur moderately in individuals from groups O and A and biochemical alterations in individuals A, B, and O [14]. Leprosy seems to be common in ORh + individuals, where changes in the blood count are greater in non-O individuals [15].

Chronic kidney disease seems to be more frequent in ORh+ blood group patients, followed by ARh+ and BRh+ , who resided in urbanized and rural areas born in the north of Angola [16]. Patients with nephrotic syndrome (NS) and sickle cell anaemia, found that the majority of the population belonged to the ORh + group, followed by patients from the ABRh+ , ARh+ , and BRh+ groups [17]. In hypertensive individuals, most patients had blood group B, blood group O, and, Rh+ [18], and the incidence of sickle cell trait was found to be high among individuals from the ORh+ and ABRh+ group [19].

It was found that studies are showing that genetic factors such as red blood cell polymorphisms and sickle cell anaemia may have influenced the severity of the disease due to *P. falciparum* infection, however, there is a lack of information about the role of host genetic factors (such as ABO/Rh blood group and sickle cell trait) in changing the blood count of patients with malaria. In this study, we investigated changes in the blood count in patients with malaria according to blood groups (ABO/Rh) and sickle cell trait admitted to a tertiary hospital in Luanda, the capital city of Angola.

## Methods

### Study design and setting

A longitudinal, prospective, and observational study was performed with 217 patients hospitalized due to malaria in Josina Machel, a tertiary Hospital, from March to August 2023. The patients were invited and freely consented to participate in the study, those patients who were unable to provide blood samples or to give their informed consent were excluded from the study population. A total of 198 patients fulfilled the inclusion criteria and were enrolled in the study. The study protocol was revised and approved by the scientific council of the Institute of Health Sciences of Agostinho Neto University (118/GD/ICISA/UAN/2021) and by the clinical management of Josina Machel Hospital (36/DPC/HJM/2023).

### Patient’s enrolment criteria and sample collection

Malaria diagnosis were performed by Josina Machel Hospital professionals using rapid malaria antigen tests (SD-Bioline Malaria AG Pf/PAN) and confirmed with microscopy technique of direct visualization of the parasite by Giemsa-stained peripheral blood thick films. Patients who presented parasitaemia less than or equal to 1000 p/mm^3^ were classified as moderate parasitaemia whilst patients who presented parasitaemia above 1000 p/mm^3^ were classified as high parasitaemia [20]. For the clinical data presented in the article, a blood sample was taken from the patients in test tubes containing EDTA (ethylenediaminetetraacetic acid) anticoagulant specific for the ABO and Rh blood group phenotyping tests and for the electrophoresis examination. Haemoglobin electrophoresis was performed using a device called Sebia brand Minicap, the Hb (E) minicab kit was developed to separate normal haemoglobins (A, F, and A2) and to detect and quantify variant haemoglobins (including S, C, E, D). Blood group determination was performed by the microplate technique, which is an agglutination test between patient serum and Anti A, Anti B, and Anti D reagents in each of the wells for phenotypic identification of blood groups (ABO and Rh) [21]. The samples were placed in three wells and the posterior was associated with anti-A, anti-B, and anti-D reagents (Immucor, Portugal).

The erythrogram and white blood cell data were evaluated on the admission of patients before starting treatment, complete blood count or haemogram was determined using the Automated Hematology Analyzer SYSMEX XT-4000i (Sysmex Europe SE, Germany). For the erythrogram data, haemoglobin (Hb), red blood cell count (RBCs), haematocrit (Hct), mean corpuscular volume (MCV), mean corpuscular haemoglobin (MCH), and mean corpuscular haemoglobin concentration (MCHC) were evaluated. For the leukocyte count data, the lymphocytes, platelets, neutrophils, and leukocyte counts were evaluated [22]. The study did not include monocyte count, eosinophil count, neutrophil-to-lymphocyte ratio (NLR), and monocyte-to-lymphocyte ratio (MLR) due to the devices that had some problems in reading these exams in some of the patients, they did not obtain data from all patients included in the study, these data were not analyzed in the study because they were not complete for all patients. In cases where the reference values of blood cell count components were different between men and women, the reference values were adjusted for the whole group based on the minimum value for women and the maximum value for men, in addition, adjustment was performed according to the references according to the specific characteristics of the Angolan population, as can be seen in the presentation of the results. All blood count results were classified as altered and clinically significant when they were higher or lower than 10% of the reference value for the test.

### Statistical analysis

Descriptive statistics were calculated using the statistical program SPSS v20.0 (IBM SPSS Statistics, USA), and the results presented in graphs were developed using Sigmaplot 12.0 (Systat Software, Inc.). The descriptive analysis was presented with frequencies and percentages. The normal distribution of data was presented as mean and standard deviation (SD). The Chi-square (X^2^) test was used to assess the relationship between categorical variables. All reported p-values are two-tailed and deemed significant when p < 0.05.

## Results

### Clinical data and distribution of ABO/Rh blood groups and sickle cell trait

Clinical data are presented in Table 1, where it was found that in terms of age groups, most patients included in the study were young (75.8%, n = 150/198) aged between 20 and 40 years. It was in this group where only individuals with the ARh(−) blood group and it was in this group where the majority of patients with sickle cell anaemia (75%) and with sickle cell trait (89.2%) were identified. As for gender, it was found that women constituted the majority in this study group (61.6%, n = 122/198) and they also represented the majority of patients with sickle cell anaemia (75%) and with the sickle cell trait (78.4%), in all blood groups women had 50% or more, in groups ABRh (+), ARh(+) and ORh(+), men represented less than 40% of the studied population. In assessing the degree of parasitaemia, it was noticed that most patients had low parasitaemia on hospital admission (less than 51 parasites/mm3) and among individuals with sickle cell anaemia, 93.8% had low parasitaemia, high parasitaemia was observed mostly in individuals of the ABRh(+), BRh(+) and ORh(+) group, which presented a percentage ranged between 25% and 38%. As for the clinical condition, most patients had a clinical picture considered moderate (53.5%, n = 106/198) and this picture was also verified concerning sickle cell trait, but it was found that 46.5% of ABRh patients (+) had a severe clinical picture, patients in the ARh(+), BRh(+) and ORh(−) group also had a severe clinical picture equal to or greater than 20%. Statistical analysis showed no statistical relationship between age group, gender, parasitaemia, and clinical condition with blood groups (p < 0.05). There was no relationship between age, parasitaemia, and clinical condition with sickle cell trait (p < 0.05), but there was a relationship between gender and sickle cell trait (p > 0.05).

**Table 1:** Clinical data and distribution of ABO/Rh blood groups and sickle cell trait

Clinical data	Blood groups						Total	Sickle cell trait		
	ABRh+	ARh−	ARh+	BRh+	ORh−	ORh+		AA	AS	SS
	13 (6.6)	4 (2.0)	49 (24.7)	42 (21.2)	5 (2.5)	85 (42.9)	198 (100)	145 (73.2)	37 (18.7)	16 (8.1)
Age groups (years olds)										
Teenager (< 20)	2 (15.4)	0 (0.0)	6 (12.2)	4 (9.5)	0 (0.0)	9 (10.6)	21 (10.6)	18 (12.4)	0 (0.0)	3 (18.8)
Young (20–40)	10 (76.9)	4 (100.0)	40 (81.6)	31 (73.8)	3 (60.0)	62 (72.9)	150 (75.8)	105 (72.4)	33 (89.2)	12 (75.0)
Adult (41–60)	0 (0.0)	0 (0.0)	2 (4.1)	4 (9.5)	2 (40.0)	9 (10.6)	17 (8.6)	15 (10.3)	2 (5.4)	0 (0.0)
Old (> 60)	1 (7.7)	0 (0.0)	1 (2.0)	3 (7.1)	0 (0.0)	5 (5.9)	10 (5.1)	7 (4.8)	2 (5.4)	1 (6.2)
Pearson Chi-Square (X^2^)	*p* = *0.601*							*p* = *0.181*		
Gender(F/M)										
Female	10 (76.9)	2 (50.0)	30 (61.2)	22 (52.4)	3 (60.0)	55 (64.7)	122 (61.6)	81 (55.9)	29 (78.4)	12 (75.0)
Male	3 (23.1)	2 (50.0)	19 (38.8)	20 (47.6)	2 (40.0)	30 (35.3)	76 (38.4)	64 (44.1)	8 (21.6)	4 (25.0)
Pearson Chi-square (X^2^)	*p* = *0.641*							***p* = *0.022***		
Parasitaemia(parasite/mm^3^)										
Low (< 51)	8 (61.5)	4 (100.0)	34 (69.4)	25 (59.5)	5 (100.0)	56 (65.9)	132 (66.7)	91 (62.8)	26 (70.3)	15 (93.8)
Moderate (51–1000)	1 (7.7)	0 (0.0)	6 (12.2)	1 (2.4)	0 (0.0)	7 (8.2)	15 (7.6)	11 (7.6)	4 (10.8)	0 (0.0)
High (> 1000)	4 (30.8)	0 (0.0)	9 (18.4)	16 (38.1)	0 (0.0)	22 (25.9)	51 (25.8)	43 (29.7)	7 (18.9)	1 (6.2)
Pearson Chi-Square (X^2^)	*p* = *0.317*							*p* = *0.100*		
Clinical condition (hospital admission)										
Mild clinical	2 (15.4)	0 (0.0)	13 (26.5)	9 (21.4)	2 (40.0)	20 (23.5)	46 (23.2)	30 (20.7)	11 (29.7)	5 (31.2)
Moderate clinical	5 (38.5)	4 (100.0)	25 (51.0)	21 (50.0)	2 (40.0)	49 (57.6)	106 (53.5)	77 (53.1)	21 (56.8)	8 (50.0)
Severe clinical	6 (46.2)	0 (0.0)	11 (22.4)	12 (28.6)	1 (20.0)	16 (18.8)	46 (23.2)	38 (26.2)	5 (13.5)	3 (18.8)
Pearson Chi-Square (X^2^)	*p* = *0.435*							*p* = *0.430*		

The bold number was statistically significant in the Chi-square test (X^2^) or independent-sample T-tests (p < 0.05)

### Condition of work and ABO/Rh blood groups and sickle cell trait

**Fig. 1:**
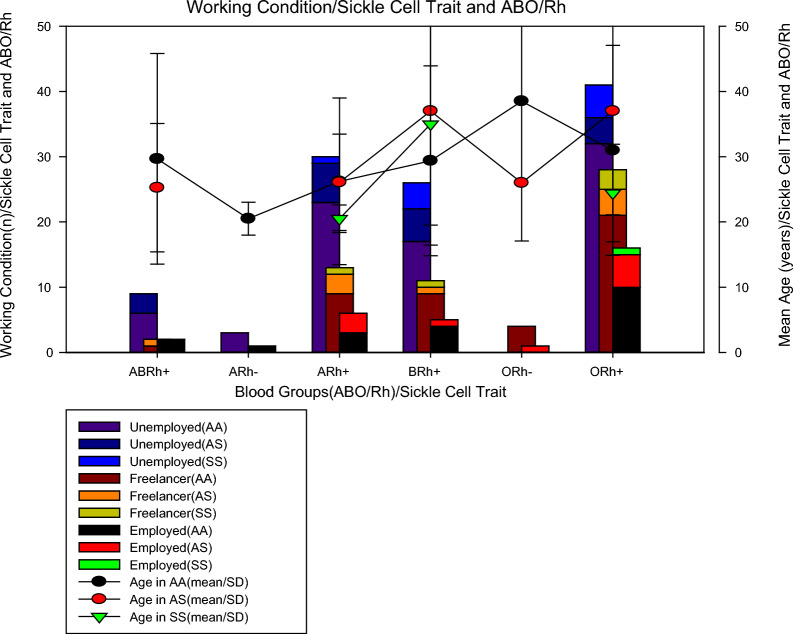
Condition of work and ABO/Rh blood groups and sickle cell trait

In Fig. 1, it can be seen that of the 198 patients studied, regarding blood groups, 13 (6.6%) were from the ABRh(+) group, 4 (2.0%) were from the ARh(−) group, 49 (24.7%) were from the ARh(+) group, 42 (21.2%) were from the BRh(+) group, 5 (2.5%) were from the ORh(−) group and 85 (42.9%) were from the ORh(+) group. As for the sickle cell trait, we found that 145 (73.2%) were AA, 37 (18.7%) were AS and 16 (8.1%) were SS. Of the 16 (8.1%) patients with sickle cell anaemia, 2 were in the anaemia+) group, 5 in the BRh(+) group, and 9 in the ORh(+) group, no Rh(-) patients with sickle cell trait were found. One piece of information that aroused the most interest was to verify the work status of patients with sickle cell anaemia and it was found that among individuals with sickle cell trait, most were unemployed (62.5%, n = 10/16), others were self-employed (31, 25%, n = 5/16) and only 6.25% (1/16) work formally. To rule out the possibility that unemployment was due to age, it was calculated the mean age of the individuals studied in those studied blood groups and sickle cell traits and it was noted that all groups of individuals with sickle cell anaemia belonging to the ARh(+) groups, BRh(+) and ORh(+) had a mean age greater than 20 years old, which means that age was not the limiting factor, more possibly the health condition.

### Evaluation of the erythrogram according to the ABO/Rh blood groups and sickle cell trait

In evaluating the erythrogram (Table 2), most patients were found to have normal haemoglobin values (above 10 mg/dL), however, it was found that all patients in the ARh(−) blood group had low haemoglobin, while in the other blood groups individuals with low haemoglobin represented percentages below 32%, while patients with sickle cell anaemia were the only ones in which 50% had low haemoglobin, in normal individuals and with sickle cell trait the percentages of individuals with low haemoglobin were less than 25%. In the evaluation of haematocrit, it was found that individuals with hematocrit that individuals with haematocrit results below the reference values (39–55%) giving a margin of 10% more than the reference values, were mostly from the ARh(−) blood group in 75% and BRh(+) in 50%, it was found that among individuals with sickle cell anaemia, individuals with low haematocrits represented about 62% of the population, while for normal individuals (AA) and with sickle cell trait (AS) these percentages were below 41%.

**Table 2:** Evaluation of the Erythrogram according to the ABO/Rh Blood Groups and Sickle Cell Trait

Erythrogram	Blood groups						Total	Sickle cell trait		
	ABRh+	ARh−	ARh+	BRh+	ORh−	ORh+		AA	AS	SS
	13 (6.6)	4 (2.0)	49 (24.7)	42 (21.2)	5 (2.5)	85 (42.9)	198 (100)	145 (73.2)	37 (18.7)	16 (8.1)
Haemoglobin (Hb)										
Low (≤ 9.9 g/dL)	3 (23.1)	4 (100.0)	12 (24.5)	13 (31.0)	1 (20.0)	16 (18.8)	49 (24.7)	36 (24.8)	5 (13.5)	8 (50.0)
Normal (10–17 g/dL)	10 (76.9)	0 (0.0)	37 (75.5)	29 (69.0)	4 (80.0)	69 (81.2)	149 (75.3)	84 (75.2)	32 (86.5)	8 (50.0)
Pearson Chi-Square (X^2^)	***p* = *0.012***							***p* = *0.018***		
Haematocrit (Hct)										
Low (≤ 31%)	5 (38.5)	3 (75.0)	21 (42.9)	21 (50.0)	1 (20.0)	28 (32.9)	79 (39.9)	58 (40.0)	11 (29.7)	10 (62.5)
Normal (31.5–50%)	8 (61.5)	1 (25.0)	28 (57.1)	21 (50.0)	4 (80.0)	57 (67.1)	119 (60.1)	87 (60.0)	26 (70.3)	6 (37.5)
Pearson Chi-Square (X^2^)	*p* = *0.254*							*p* = *0.083*		
Mean corpuscular volume (MCV)										
Low (≤ 70 fL)	3 (23.1)	4 (100.0)	17 (34.7)	14 (33.3)	1 (20.0)	25 (29.4)	64 (32.3)	42 (29.0)	14 (37.8)	8 (50.0)
Normal (71–100 fL)	10 (76.9)	0 (0.0)	32 (65.3)	28 (66.7)	4 (80.0)	60 (70.6)	134 (67.7)	103 (71.0)	23 (62.2)	8 (50.0)
Pearson Chi-Square (X^2^)	*p* = *0.084*							*p* = *0.170*		
Mean corpuscular haemoglobin (MCH)										
Low (≤ 24 pg)	5 (38.5)	4 (100.0)	15 (30.6)	10 (24.4)	1 (20.0)	24 (28.6)	59 (30.1)	39 (27.3))	14 (37.8)	6 (37.5)
Normal (24.1–32.6 pg)	8 (61.5)	0 (0.0)	34 (69.4)	31 (75.6)	4 (80.0)	60 (71.4)	137 (69.9)	104 (72.7)	23 (62.2)	10 (62.5)
Pearson Chi-Square (X^2^)	*p* = *0.058*							*p* = *0.366*		
Mean corpuscular haemoglobin concentration (MCHC)										
High (≥ 39.7)	13 (100.0)	4 (100.0)	47 (95.9)	38 (90.5)	5 (100.0)	81 (95.3)	188 (94.9)	138 (95.2)	35 (94.6)	15 (93.8)
Normal (27.9–39.6%)	0 (0.0)	0 (0.0)	2 (4.1)	4 (9.5)	0 (0.0)	4 (4.7)	10 (5.1)	7 (4.8)	2 (5.4)	1 (6.2)
Pearson Chi-Square (X^2^)	*p* = *0.694*							*p* = *0.946*		

The bold number was statistically significant in the Chi-square test (X^2^) or independent-sample T-tests (p < 0.05)

The evaluation of the mean corpuscular volume (MCV), verified that the majority (67.7%, n = 134/198) of the individuals presented normal values (between 71 and 100 fL), however, all the patients of the blood group ARh(-) showed low MCV, while in the other blood groups individuals with low haemoglobin represented percentages below 38%, patients with sickle cell anaemia were the only ones in which 50% of them had low MCV, in normal individuals and with trait sickle cell, the percentages of individuals with low MCH were less than 38%.

The assessment of mean corpuscular haemoglobin (MCH), found that most individuals (68.9%, n = 137/198) of the patients studied who had normal MCH results (24.1–32.6 pg), all (100%) individuals from the ARh(-) blood group had MCH below the reference values. In other groups, the percentage of patients with low MCH did not exceed 38%, and the MCH did not show many differences between individuals with sickle cell anaemia, or normal individuals (AA) and individuals with sickle cell trait (AS), since the percentages for all cases were between 37.8% to 27.3%.

The assessment of mean corpuscular haemoglobin concentration (MCHC), showed that regardless of blood group or sickle cell trait, most patients (above 90% in all groups) had high MCHC (above 39.6 mg/dL). Statistical analysis showed a relationship between haemoglobin values and ABO/Rh blood groups (p < 0.05), but there was no relationship between haematocrit, VCM, MCH, and MCHC values in blood groups (p > 0.05). Also, there was a relationship between haemoglobin values and sickle cell trait (p < 0.05), but there was no relationship between haematocrit, VCM, MCH, and MCHC values with sickle cell trait (p > 0.05).

### Erythrogram parameters in ABO/Rh blood groups and sickle cell trait

**Fig. 2:**
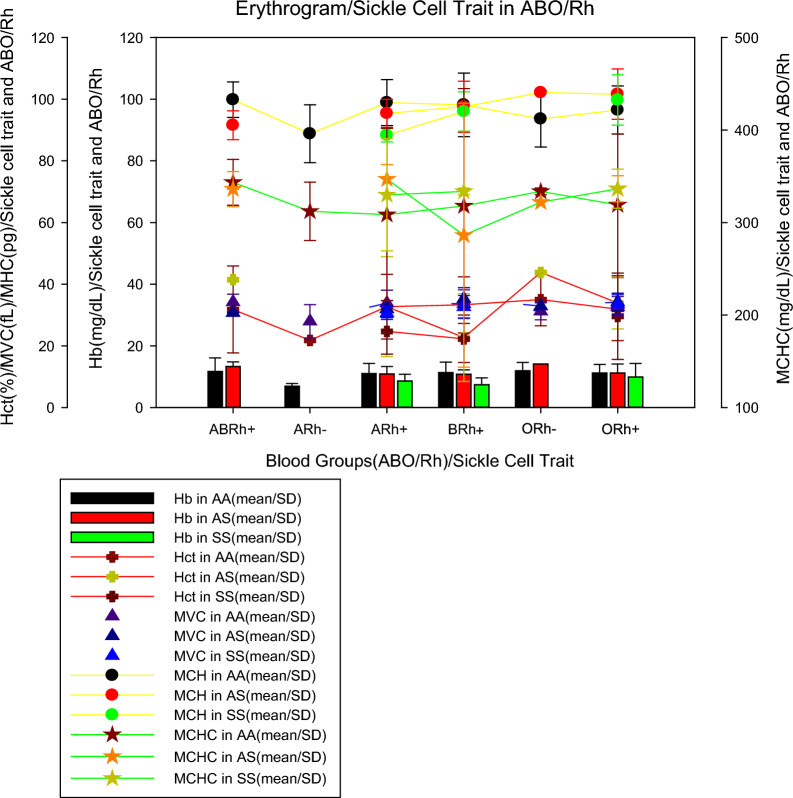
Erythrogram parameters in ABO/Rh blood groups and sickle cell trait

Figure 2 shows the mean values of the erythrogram parameters (mean ± SD) by blood group and sickle cell trait, where it can be seen that individuals from the ARh(−) blood group, without sickle cell trait (AA), had lower haemoglobinin values (6.95 ± 1.7 mg/dL) than individuals with sickle cell anaemia in groups A (8.6 ± 2.2 mg/dL), B (7.4 ± 2.2 mg/dL) and O (9, 9 ± 4.4 mg/dL) all Rh(+) as previously described, ARh(−) patients showed the lowest mean erythrocyte values (21.8 ± 0.1%), MCV (23.3 ± 4.5 fL), MCH (74.0 ± 7.0 pg) except for MCHC which was increased in all patients, including patients with RA(−). For sickle cell traits, we noticed that individuals with sickle cell anaemia (AA) belonging to the ARh(+), BRh(+), and ORh(+) groups had an average erythrocyte smaller than normal (AA) and heterozygous (AS) individuals, however, for tests such as VCM, MCH, and MCHC, in most cases, individuals with sickle cell anaemia showed values close to or higher than normal (AA) and heterozygous (AS) individuals.

### Evaluation of the leucocyte count according to the ABO/Rh blood groups and sickle cell trait

The evaluation of the leucocyte count (Table 3), showed that more than half of the patients had normal leukocyte (WBC) values (1–4.8 × 10^9^/L), however, more than 30% of patients from all blood groups had altered (increased) WBC, except patients in the ARh(−) group, all had normal WBC values, for sickle cell trait, it was found that more in all groups (AA, AS, SS) more than 40% of individuals who had altered (increased) WBC values. The evaluation of neutrophils (NEUT) found that a little more than half of the patients had NEUT results between the reference values (1.8–7.7 × 10^9^/L), patients of the ABRh(+) blood group, ARh(−) and Brh(+) presented alterations in neutrophils (low) ≤ 50%. Among individuals with sickle cell anaemia, patients with altered NEUT (low) represented about 50% of the population, while for normal individuals (AA) and with sickle cell trait (AS) these percentages were below 46%.

**Table 3:** Evaluation of the Leucocyte count according to the ABO/Rh blood groups and sickle cell trait

Leucocyte count	Blood groups						Total	Sickle cell trait		
	ABRh+	ARh−	ARh+	BRh+	ORh−	ORh+		AA	AS	SS
	13 (6.6)	4 (2.0)	49 (24.7)	42 (21.2)	5 (2.5)	85 (42.9)	198 (100)	145 (73.2)	37 (18.7)	16 (8.1)
Leukocytes (WBC)										
Changed (≤ 0.8 or ≥ 5.3 × 10^9^/L)	5 (36.5)	0 (0.0)	22 (44.9)	18 (42.9)	3 (60.0)	39 (45.9)	87 (43.9)	61 (42.1)	20 (54.7)	6 (43.9)
Normal (0.9–5.2 × 10^9^/L)	8 (61.5)	4 (100.0)	27 (55.1)	24 (57.1)	2 (40.0)	46 (55.1)	111 (56.1)	84 (57.9)	17 (45.9)	10 (56.1)
Pearson Chi-Square (X2)	*p* = *0.366*							*p* = *0.551*		
Neutrophils(NEUT)										
Changed (≤ 1.5 or ≥ 8.5 × 10^9^/L)	8 (61.5)	3 (75.0)	17 (34.7)	21 (50.0)	1 (20.0)	32 (37.6)	82 (41.4)	57 (39.3)	17 (45.9)	8 (50.0)
Normal (1.6–8.4 × 10^9^/L)	5 (38.5)	1 (25.0)	32 (65.3)	21 (50.0)	4 (80.0)	53 (62.4)	116 (58.6)	88 (60.7)	20 (54.1)	8 (50.0)
Pearson Chi-Square (X2)	*p* = *0.176*							*p* = *0.587*		
Lymphocytes(LINF)										
Changed (≤ 0.8 or ≥ 5.3 × 10^9^/L)	9 (69.2)	1 (25.0)	34 (69.4)	26 (61.9)	5 (100.0)	65 (76.5)	140 (70.4)	97 (66.9)	31 (83.8)	12 (75.0)
Normal (0.9 to 5.2 × 10^9^/L)	4 (30.8)	3 (75.0)	15 (30.6)	16 (38.1)	0 (0.0)	20 (23.5)	58 (29.3)	48 (33.1)	6 (16.2)	4 (25.0)
Pearson Chi-Square (X2)	*p* = *0.122*							*p* = *0.105*		
Platelets(PTL)										
Changed (≤ 165.000 or ≥ 495.000/mm^3^)	6 (46.2)	4 (100.0)	34 (69.4)	28 (69.4)	4 (80.0)	55 (64.7)	131 (66.2)	102 (70.3)	24 (64.9)	5 (31.2)
Normal (165.000–495.000/mm^3^)	7 (53.8)	0 (0.0)	15 (30.6)	14 (30.6)	1 (20.0)	30 (35.3)	67 (33.8)	43 (29.7)	13 (35.1)	11 (68.8)
Pearson Chi-Square (X2)	*p* = *0.402*							***p* = *0.007***		

The bold number was statistically significant in the Chi-square test (X^2^) or independent-sample T-tests (p < 0.05)

The lymphocyte evaluation (LINF) verified that the majority (70.4%, n = 140/198) of the individuals presented values above the reference values (1 to 4.8 × 10^9^/L), however, in all blood groups studied there was an alteration (increase) in the leukocyte value in more than 60%, however, among patients in the ARh(−) group, these alterations occurred in only 25% of the patients, for the sickle cell trait, it was found that in all groups (AA, AS, SS) changes were greater than 66%. In the assessment of platelets (PTL), it was discovered that in the majority ty (64.2%, n = 131/198) of the patients studied who had PTL results below the reference values (150,000 and 450,000 mm^3^), in all groups the reduction was greater than 46% of platelets in all (100%) of the individuals of the different groups, it was verified for sickle cell trait, individuals with sickle cell anaemia (SS), more than 60% had normal PTL, while in the other groups, this percentage did not exceed 36%. Statistical analysis showed no relationship between WBC, NEUT, LINF, and PTL values with blood groups (p > 0.05). There was no statistically significant relationship between WBC, NEUT, and LINF values with sickle cell trait (p > 0.05), but there was a relationship between PTL and sickle cell trait (p < 0.05).

**Fig. 3:**
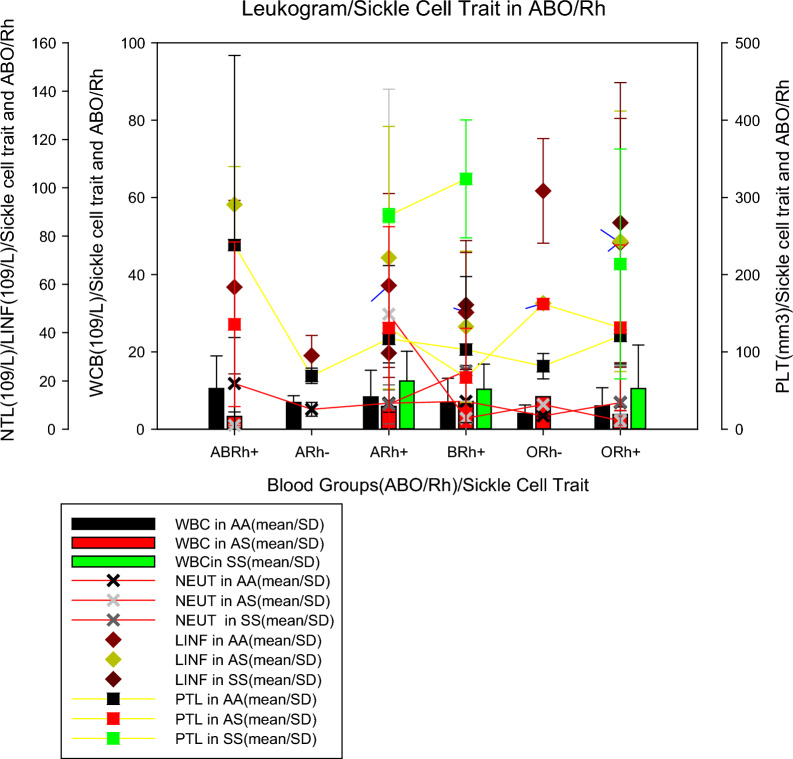
Leucogram parameters in ABO/Rh blood groups and sickle cell trait

Figure 3 shows the mean values of the leucocyte count parameters (mean ± SD) by blood group and sickle cell trait. This shows that individuals from the blood group ABRh(+) with sickle cell trait (AS), ORh(-) without sickle cell trait (AA) and ORh(+) with sickle cell trait showed lower WBC values (3.28 ± 1.19 10^9^/L, 4.05 ± 2.24 10^9^/L and 3.76 ± 1.98 10^9^/L, respectively). The mean NEUT in individuals with sickle cell trait (AS) in the ABRh(+) group and without sickle cell trait (AS) in the ARh(−) and ORh(−) groups were lower (1.60 ± 0.41 10^9^/L, 8.25 ± 2.90 10^9^/L and 5.48 ± 0.35 10^9^/L, respectively) when compared to individuals of other blood groups. The mean number of lymphocytes in individuals with ARh(−) without trait sickle cell and ARh(+) with sickle cell anaemia were the lowest (3.33 ± 3.65 10^9^/L and 3.78 ± 2.58 10^9^/L) when compared to individuals from other groups. Mean PLT in individuals without trait sickle cell(AS) from the ARh(−) and ORh(−) groups were lower (69.0 ± 10.0 10^9^/L and 81.5 ± 16.34 10^9^/L, respectively) when compared to individuals from other groups.

## Discussion

In the present study, the O blood group represented about 46% of the entire population, followed by groups A (26.7%), B (21.2), and AB (6.6%). Regarding the sickle cell trait, AS and SS represented 18.7% and 8.1%, respectively. Similar data were observed in a study carried out in Nigeria, where group O was the most frequent (47.7%), followed by blood group B (25.2%), A (22.5%), and AB (4.6%), also, the frequencies of HbAS and HbAC were 14.4% and 5.8%, respectively [23]. In the present study, women were the majority (68.6%) in all blood groups, except for the ARh(-), and also represented 75% of the SS individuals. A statistical relationship was observed between sickle cell trait and gender (p < 0.05), which seems to be similar to that reported in Ghana with children, where blood group O was the most prevalent (41.4%), followed by blood groups A (29.6%) and B (23.3%), while AB (5.7%) had the lowest frequency. The authors also showed that the prevalence rates of sickle cell trait (HbAS and HbSC) and sickle cell disease (HbSS) were 17.5% and 0.5%, respectively [25].

High parasitaemia was mostly frequent in individuals with ABRh(+) and BRh(+) blood groups representing more than 30%. The ARh(+) blood group presents the highest number of severe cases of malaria (46.2%), followed by the BRh (28.6%) and ARh (22.4%). However, both for high parasitaemia and severe malaria, individuals with AS and SS had a lower prevalence than AA individuals, which corroborates the Nigerian study where was showed that HbAS was associated with a reduced risk of severe malaria [OR = 0.46 (95% CI 0.27–0.77)]. Moreover, among individuals with severe malaria, HbAS was associated with significantly lower parasite densities. The protective effect of blood group O was demonstrated with a low risk of severe malaria [OR = 0.74 (95% CI 0.56–0.97)]. Blood group B was associated with an increased risk of severe malaria [OR = 1.63 (95% CI 1.12–2.38)] [23]. The study results found (Table 1) differ from a study conducted in Nigeria, which showed that individuals with sickle cell disease (HbSS, HbSC) had the highest prevalence of malaria parasitaemia and severe malaria. In addition, individuals with blood group O also had a higher prevalence of malaria parasitaemia, but a lower prevalence of severe malaria when compared with non-O blood groups, which confirmed the protective role of the O antigen in impairing the formation of rosettes and the vascular cytoadhesion of parasitized red blood cells, making it more susceptible to malaria infection than non-O antigens, but less susceptible to severe disease [24].

In the present study (Fig. 1), It was found that individuals with sickle cell anaemia were only from the ARh(+), BRh(+), and ORh(+) groups, with an average age of over 20 years old (Table 1). A study conducted with 300 pregnant women from Nigeria found that 73.7% were HbAA, 22.3% were HbAS, 3.7% were HbAC and 0.3% were HbSC, with HbAA being more prevalent among subjects in group O followed by A, B, and AB, but HbAS was more prevalent among group A followed by B, O, and AB while HbAC was more prevalent between groups A and O, followed by B and AB, whereas the prevalence of HbSC was concentrated in the blood group and even so there were no homozygous haemoglobinopathies S and C [26, 27].

The present study (Table 2) found a relationship between the haemoglobin values with the blood type and with the sickle cell trait (p < 0.05), where all individuals in the ARh(−) group showed alterations and individuals in the group ORh(+) had lower percentages of low haemoglobin (18.8%), however, more than 50% of SS individuals had low haemoglobin. A study carried out in Cameroon showed that the mean parasite density was significantly lower among children with SCD than among children without SCD (22,875 vs 57,053 parasites/µL, p = 0.002), where the mean haemoglobin concentration was lower in SCD compared to non-SCD (5.7 g/L vs 7.4 g/L, p ≤ 0.001) and the mean haemoglobin concentration was significantly lower among patients with SCD in the admission compared to patients without SCD (5.7 g/dL vs 7.4 g/dL, p < 0.001) [28].

In the present study, it was not observed a relationship between blood groups and SCD (Table 2), however, the blood group ARh(−) showed a different behavior from all blood groups in the results of the erythrogram, and similar data were recorded in a study carried out in Nigeria among patients with malaria, The haemoglobin (Hb), [median (IQR); 7.3 (1.3), p = 0.001], haematocrit (HCT), [median (IQR); 26.4 (4.4), p = 0.009], red blood cells (RBC), [median (IQR); 3.2 (1.7), p = 0.048] were markedly reduced in HbSS, however, red cell distribution with (RDW) [median (IQR); 14.9 (3.3), p = 0.030] increased in malaria-infected children with HbSS and severe anaemia was higher in HbSS (23.1%), followed by HbAA (8.6%) and HbAS (7.1%) [23]. Another study found that blood group B was associated with an increased risk of severe malaria [OR = 1.638 (95% CI 1.128–2.380)] and after adjusting for age, gender, haematocrit, parasite density, and Hb genotype, we confirm from this large study in Nigerian children that the greater protective effect of the heterozygous sickle cell state against cerebral malaria and severe malarial anaemia) [11, 12].

Individuals from the ARh(−) blood group, without sickle cell trait (AA), had lower haemoglobin values (6.95 ± 1.7 mg/dL) than individuals with sickle cell anaemia from groups A (8.6 ± 2.2 mg/dL), B(7.4 ± 2.2 mg/dL) and O (9.9 ± 4.4 mg/dL). We observed that all Rh(+) patients with sickle cell anaemia belonging to the ARh(+), BRh(+), and, ORh(+) had a mean number of erythrocytes smaller than normal (AA) and heterozygous (AS) individuals (Fig. 2). It seems that the very condition of infection by *P. falciparum* can promote haematological abnormalities due to the life cycle of the parasite. Previous studies concluded that haematological markers for anaemia, that is haemoglobin, haematocrit, and red blood cell count were higher in those infected with *P. falciparum* malaria, as well as the distribution of red blood cells, MCV, MCH, and MCHC also differ when comparing individuals with and without infection [23].

Leucocyte count (64.2%, n = 131/198) of the patients studied had platelets (PTL) below the reference values (150,000 and 450,000 mm^3^) and there was a significant relationship between PTL and sickle cell trait (p < 0.05). A previous study conducted in Cameroon showed that the mean PLT count was significantly higher in patients with SCD compared to patients without SCD (274,014 vs 177,668 10^9^/L; p < 0.001) and that the mean white blood cell count was lower in patients with sickle cell disease compared to patients without sickle cell disease (20.3 vs 9.8 × 10^9^/L), although this difference was not statistically significant (p = 0.394) [28]. On the other hand, a study carried out in Nigeria, showed no differences in PLT count (p = 0.399), therefore, no severe thrombocytopenia between genotypes and identified that leukocytosis was higher in HbSS (69.2%), HbAS (42%) and HbAA (31%) [11].

It was noticed that individuals from the blood group ABRh(+) with sickle cell trait (AS), ORh(−) without sickle cell trait (AA), and ORh(+) with sickle cell trait had lower WBC values, as well as the mean NEUT in individuals with sickle cell trait(AS) from the ABRh(+) group and without sickle cell trait(AS) from the ARh(−) and ORh(−) groups were lower when compared to individuals of other blood groups (Fig. 3). A study conducted in Nigeria also reported that the mean concentration of WBC, lymphocytes, monocytes and granulocyte was lower in individuals with SCD compared to individuals without SCD. Moreover, other studies revealed that PLT counts were lower in the malaria-infected group [median (IQR), 236 (129.5) vs. uninfected group [median (IQR), 278 (112.8), p = 0.001] [11]. Previous studies reported that monocytosis is associated with hemolysis and inflammation in sickle cell anaemia, however, the influence of carrying different HBB genotypes (6GAG > 6G TG) on the severity of major haematological abnormalities and correlation of haematological parameters with HBB genotypes (6G AG > 6G TG) [12,29]. Future studies exploring the demographic, genetic, clinical, and laboratory determinants related to sickle cell anaemia should be carried out in patients with Malaria in Angola.

## Conclusion

With the results of the study, it can be concluded that individuals from the ORh + blood group, followed by the ARh(+) and BRh(+) groups seem to be the majority as well in malaria infection among Angolan patients. However, the ARh(−) group seems to be the one that most suffers the impact of the disease both in the erythrocyte and leukocyte responses, even in the low parasitaemia and moderate disease, which is different in the ABRh(+) group, which seems to be the most susceptible to severe disease and which individuals with sickle cell anaemia seem to have a poor response in the replacement of the erythrocyte response and some leucocytic parameters. The study results highlight the importance of differentiated care for malaria patients according to ABO/RH blood groups and the sickle cell trait, taking into account the haemogram parameters that demonstrate how patients can react to infection regardless of the degree of parasitaemia and medical prognosis.

## Data Availability

All relevant data are within the paper.
